# Supplementary material for: Investigation of Mating Pheromone–Pheromone Receptor Specificity in Lentinula edodes
Source: Genes (Basel). 2020 May 4;11(5):506. doi: 10.3390/genes11050506 (PMC7288658; doi:10.3390/genes11050506)
Supplement: Supplementary file 1 [file genes-11-00506-s001.zip › TableS1.docx]

**Table S1. Primers used for cloning**

| Primer name | Direction | Nucleotide sequence |
| --- | --- | --- |
| Sst2 front | Forward (5` – 3`) | GTCATCGTTTAACTCCATG |
|  | Reverse (5` – 3`) | CATAAAAAAATATAGAGTGTACTAGGCTTGCCATCCTTGTTCTGG |
| Sst2 end | Forward (5` – 3`) | CCAAAGGTGTTCTTATGTAGGACATGCTTATCTCTTCGAG |
|  | Reverse (5` – 3`) | GGACGAATAAATGTGATATGCC |
| HIS3 | Forward (5` – 3`) | CCAGAACAAGGATGGCAAGCCTAGTACACTCTATATTTTTTTATG |
|  | Reverse (5` – 3`) | CTCGAAGAGATAAGCATGTCCTACATAAGAACACCTTTGG |
| Gpa1 front | Forward (5` – 3`) | GATTTGTTCGAGGAAAAGG |
|  | Reverse (5` – 3`) | CATCCAGCAAAACCTTAAAGAGGCCGGACTATTATAGGTACAATCTTGATCCGGAGC |
| Gpa1 end | Forward (5` – 3`) | CGTCAAAAATGCTAAGAAATAGAGGAACTGTATAATTAAAGTA |
|  | Reverse (5` – 3`) | CCCTGTCCTTACGTAGAAGAATC |
| TRP1 | Forward (5` – 3`) | GCTCCGGATCAAGATTGTACCTATAATAGTCCGGCCTCTTTAAGGTTTTGCTGGATG |
|  | Reverse (5` – 3`) | TACTTTAATTATACAGTTCCTCTATTTCTTAGCATTTTTGACG |
| Ste2 promoter | Forward (5` – 3`) | ATTGGGTACCGAGAAGAAGCATCCTGC |
|  | Reverse (5` – 3`) | TTAGATGGGCCCTTTTGATTCTTGGATATGGTTCTTAAC |
| Ste2 terminator | Forward (5` – 3`) | TATACTGAATTCAAAATTTACGGCTTTGAAAAAG |
|  | Reverse (5` – 3`) | TCTAGAACTAGTTACCATCATTTTATTTGTGTAC |
| KanMX6 | Forward (5` – 3`) | CTCGAGGTCGACATGGAGGCCCAGAATAC |
|  | Reverse (5` – 3`) | AATTTTGAATTCAGTATAGCGACCAGCATTC |
| RCB1-2 CDS | Forward (5` – 3`) | GGATCCATGCAAACTTCCGATCCAACG |
|  | Reverse (5` – 3`) | CCCGGGCTACCAGAATGCGCGGC |
| RCB1-4 CDS | Forward (5` – 3`) | GGATCCATGCAAGCGTCCGATCCTA |
|  | Reverse (5` – 3`) | CCCGGGCTACCAGAATGCGCGGC |
| RCB2-1 CDS | Forward (5` – 3`) | GGATCCATGGCCAATCTCGACTATC |
|  | Reverse (5` – 3`) | CCCGGGTCACGTTTGTACATCGGAAG |
