# Supplementary material for: Investigation of Mating Pheromone–Pheromone Receptor Specificity in Lentinula edodes
Source: Genes (Basel). 2020 May 4;11(5):506. doi: 10.3390/genes11050506 (PMC7288658; doi:10.3390/genes11050506)
Supplement: Supplementary file 1 [file genes-11-00506-s001.zip › TableS2.docx]

**Table S2. Primers used for real-time PCR**

| Primer name | Direction | Nucleotide sequence |
| --- | --- | --- |
| RCB1-2 RT | Forward (5` – 3`) | GATGTAGCTATCGCGGTCGG |
|  | Reverse (5` – 3`) | GGACTAATGGTACTGTTGG |
| RCB1-4 RT | Forward (5` – 3`) | GACCTCGCCATTGCTTTTGG |
|  | Reverse (5` – 3`) | GGATTGATGGCCCTATTCG |
| RCB2-1 RT | Forward (5` – 3`) | GTTTATGGCCCCTGGTATATG |
|  | Reverse (5` – 3`) | GTCTGAAATATCGGCCAAAG |
| Fus1 | Forward (5` – 3`) | GCAGAGTAAGTGTGAGGATCAG |
|  | Reverse (5` – 3`) | GGTTTAGTATACAGGAATGCATCC |
| β-tubulin (Sc) | Forward (5` – 3`) | GATGGCCACCTTCTCCGTCTTG |
|  | Reverse (5` – 3`) | GTTCAATTGGCCGGGATAACG |
| β-tubulin (Le) | Forward (5` – 3`) | GACCGTATGATGTGCACGTAC |
|  | Reverse (5` – 3`) | CACAAGATGGTTGAGGTCACC |
| Znf2 | Forward (5` – 3`) | CAGCAACACCAATCCGACCG |
|  | Reverse (5` – 3`) | GAGTGCTCCGATTACCTTTG |
| HD1 | Forward (5` – 3`) | CACTCTATCGTCGAAACTGC |
|  | Reverse (5` – 3`) | CTACCAAGTCCCTCCTCCTC |
| HD2 | Forward (5` – 3`) | CGGAGGTGCCGAAGTATAAG |
|  | Reverse (5` – 3`) | CTTATCCCGATAAAGCATCC |
| PriA | Forward (5` – 3`) | CCGTCATCGAAGTTGTTGTC |
|  | Reverse (5` – 3`) | GCACTTGCCGTTGCAAACTG |
| Clp1 | Forward (5` – 3`) | CCGAGTACTTCGCAACCAAC |
|  | Reverse (5` – 3`) | GTCGACCTGTGTTACCACGC |
