# Supplementary figures and images for: Investigation of Mating Pheromone–Pheromone Receptor Specificity in Lentinula edodes
Source: Genes (Basel). 2020 May 4;11(5):506. doi: 10.3390/genes11050506 (PMC7288658; doi:10.3390/genes11050506)

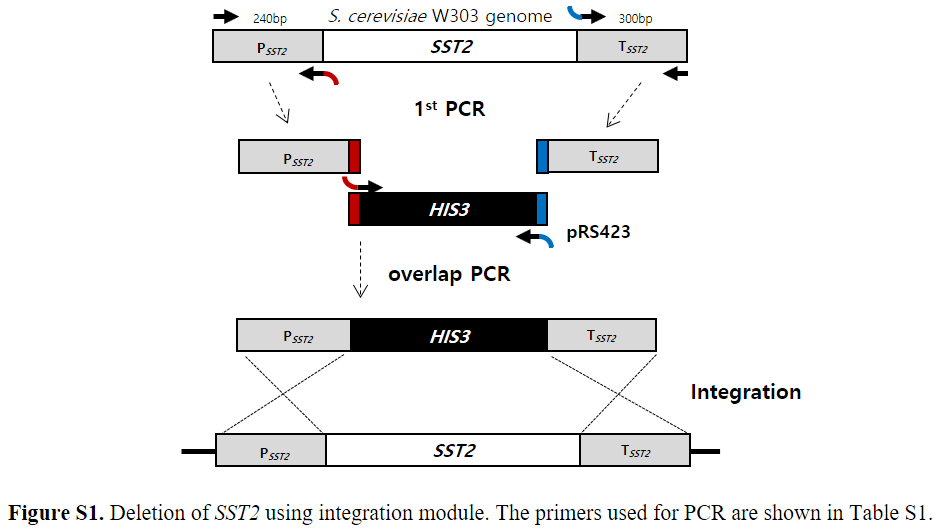

Supplement: Supplementary file 1 [file genes-11-00506-s001.zip › FigS1.TIF]

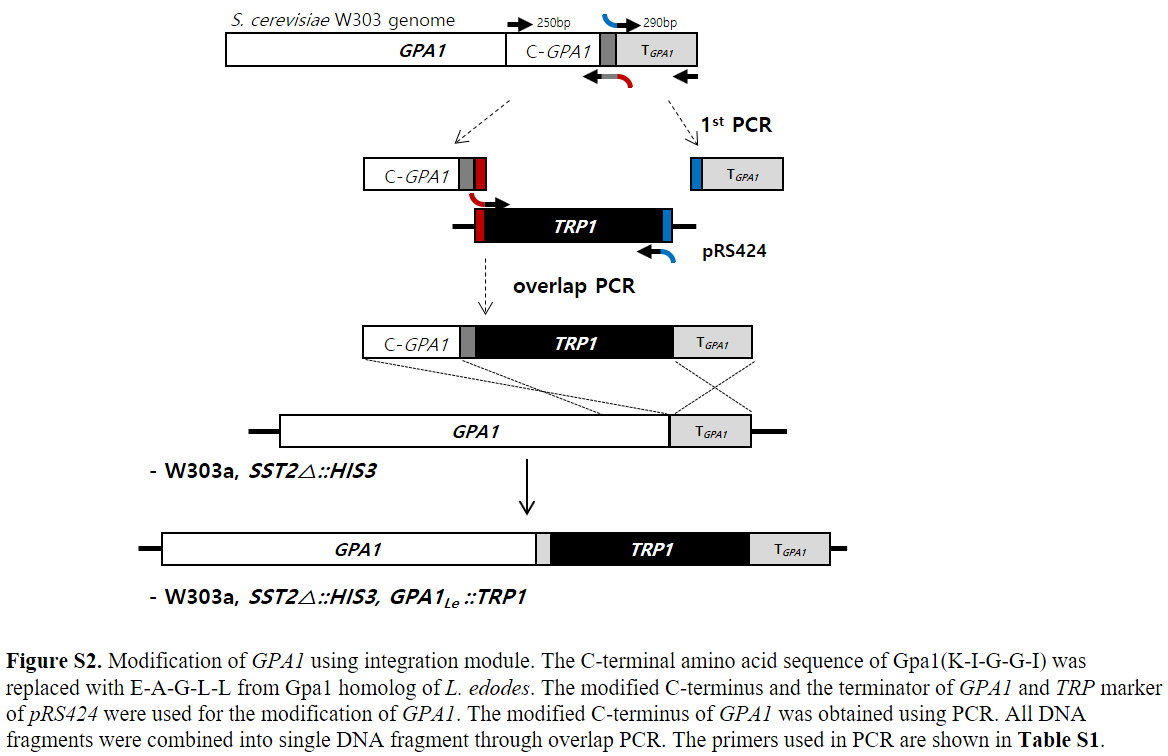

Supplement: Supplementary file 1 [file genes-11-00506-s001.zip › FigS2.TIF]

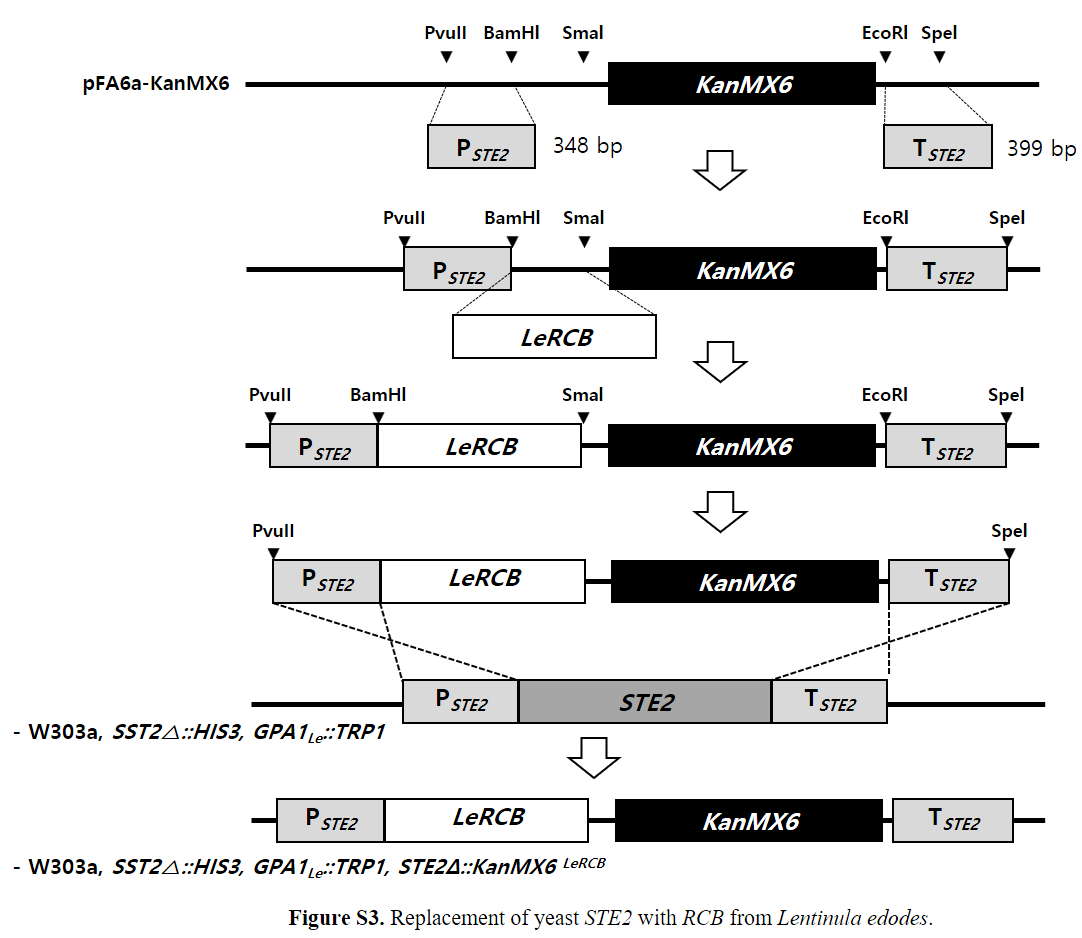

Supplement: Supplementary file 1 [file genes-11-00506-s001.zip › FigS3.TIF]

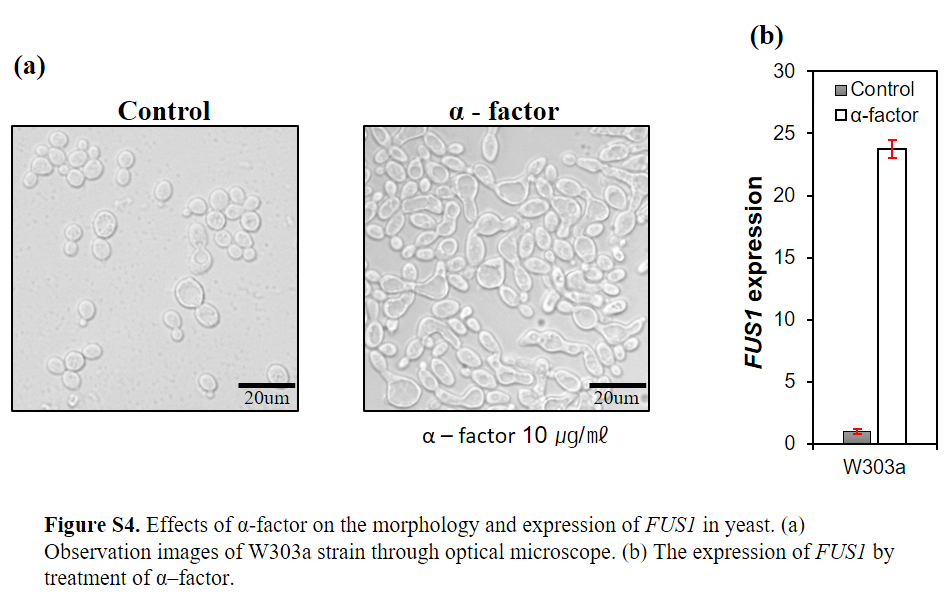

Supplement: Supplementary file 1 [file genes-11-00506-s001.zip › FigS4.TIF]

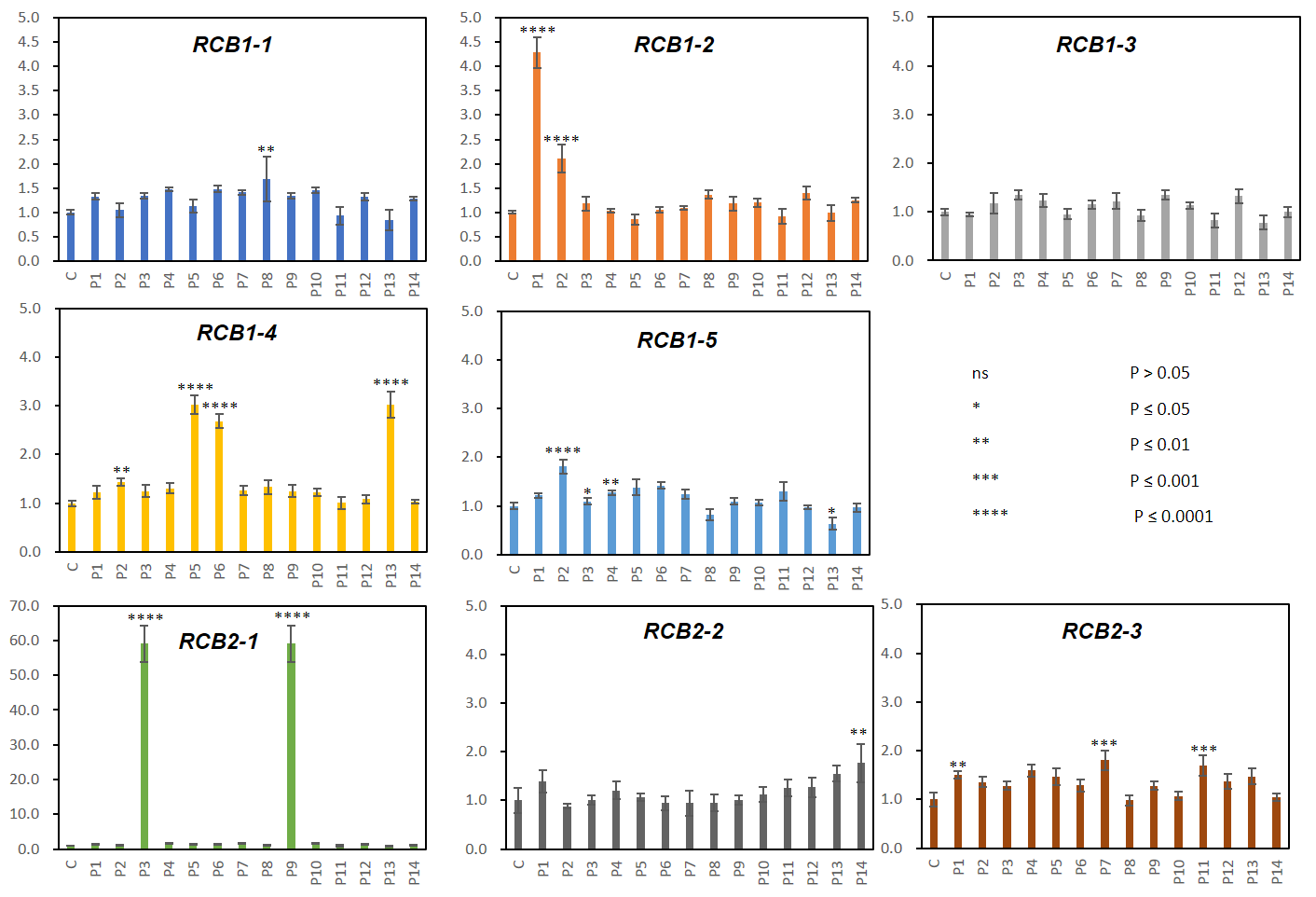

Supplement: Supplementary file 1 [file genes-11-00506-s001.zip › FigS5.TIF]
